# 3D analysis and evolution of prostate and tumor oxygenation using MRI-derived relative oxygen extraction fraction during MR-guided stereotactic body radiotherapy^☆^

**DOI:** 10.1016/j.phro.2026.101097

**Published:** 2026-10-01

**Authors:** Thomas Berger, Ariane Lapierre, Perla Zidane, Hayder Aloraibi, Samy Horn, Angela Boros, Frederic Cervenansky, Benjamin Leporq, Olivier Beuf, William Henry Nailon, Olivier Chapet

**Affiliations:** a Radiation Oncology Department, Centre Hospitalier Lyon Sud, Hospices Civils de Lyon, France; b Claude-Bernard Lyon 1 University, Lyon, France; c INSA-Lyon, Université Lyon 1, CNRS, Inserm, CREATIS UMR 5220, U1294, Lyon, France; d Department of Oncology Physics, Edinburgh Cancer Centre, Western General Hospital, Crewe Road South, Edinburgh, EH4 2XU, UK; e Institute for Imaging, Data and Communications (IDCOM), School of Engineering, University of Edinburgh, Kings Buildings, Edinburgh EH9 3BF, UK

**Keywords:** Stereotactic body radiotherapy, Prostate cancer, Hypoxia, Oxygen extraction fraction, MR-guided radiotherapy

## Abstract

**Background/Purpose:**

Tumor hypoxia is a major cause of radioresistance, motivating the development of biomarkers related to tissue oxygen physiology that can be monitored throughout treatment. MRI-derived relative Oxygen Extraction Fraction (rOEF) is a non-invasive imaging biomarker related to tissue oxygen extraction. This study characterized the temporal evolution of rOEF during MR-guided stereotactic body radiotherapy (MRgSBRT) for prostate cancer.

**Materials/Methods:**

Twenty-three prostate cancer patients treated with five-fraction MRgSBRT were analyzed. rOEF maps were acquired at treatment planning and at each treatment fraction. Average rOEF was extracted from the healthy prostate, gross tumor volume (GTV), and two gluteal muscle regions receiving either residual (in-field) or negligible (out-of-field) irradiation. Longitudinal changes in average rOEF and in the fraction of GTV voxels with rOEF >70% were quantified using patient-specific linear fits. Slopes of average rOEF in the healthy prostate were also calculated between successive imaging sessions.

**Results:**

Average rOEF decreased significantly in all irradiated regions (healthy prostate, GTV and in-field gluteal muscle), with no significant change in the out-of-field gluteal muscle. The fraction of GTV voxels with rOEF >70% also declined significantly. In the healthy prostate, rOEF decreased predominantly during the first four fractions, with the steepest median slope observed between planning and the first treatment fraction (−3.2).

**Conclusions:**

MRgSBRT was associated with a progressive reduction in rOEF confined to irradiated tissues, with the largest decrease in healthy prostate rOEF occurring after the first irradiation. These findings suggest rOEF could be a potential imaging biomarker of physiological changes associated with radiation exposure and warrant further investigation of its relationship with physiological processes and tissue hypoxia.

## Highlights

• Daily MR-linac imaging enabled 3D tracking of prostate oxygen extraction fraction.
• Irradiated prostate, tumor, and in-field muscle showed decreased oxygen extraction fraction.
• Out-of-field muscle showed no significant change in oxygen extraction fraction.
• Tumor high-oxygen extraction fraction decreased during treatment.
• Tumor oxygen extraction fraction patterns changed over the course of treatment.

## Graphical abstract

**Figure:**
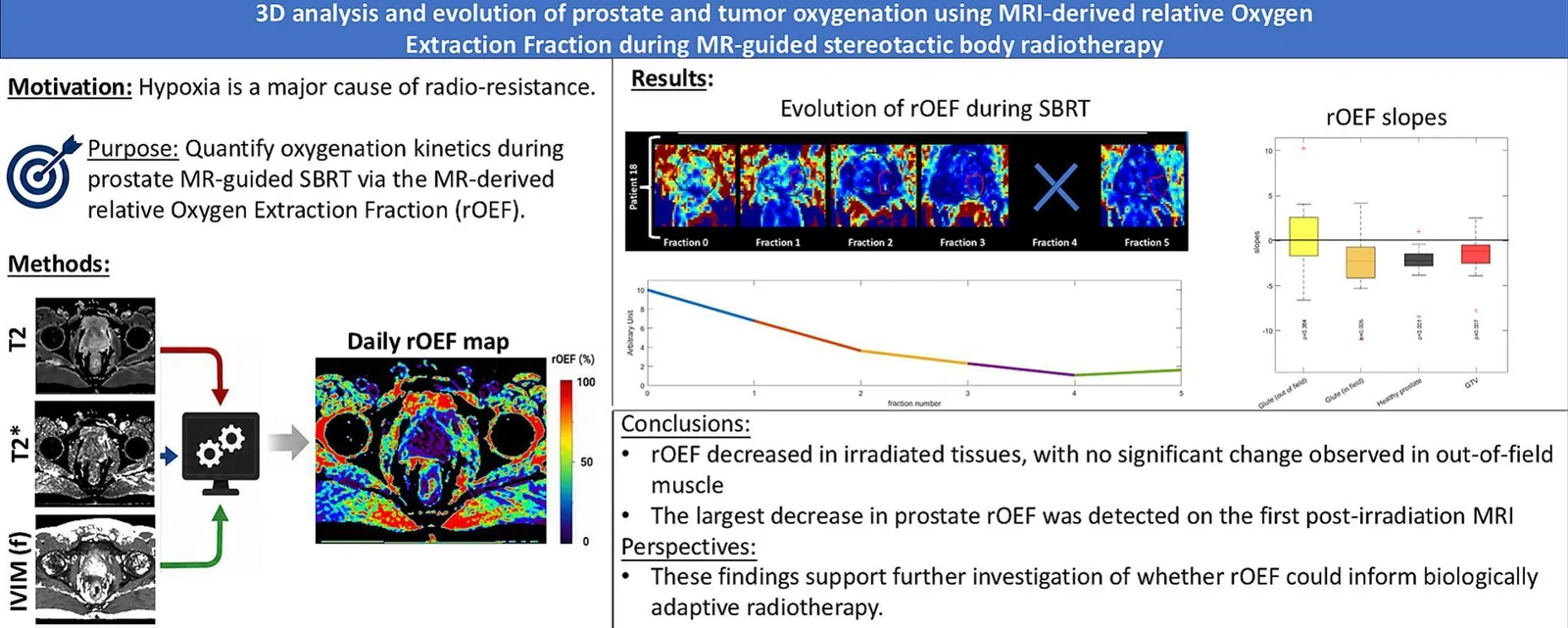

## 1 Introduction

With nearly 1.5 million newly diagnosed cases, prostate cancer was the second most common malignancy in men in 2022 [1]. External beam radiotherapy is a non-invasive effective curative treatment for localized prostate cancer. It conventionally consists of delivering the prescribed radiation dose over the course of four to eight weeks. In recent years, clinical practice has shifted towards much shorter fractionation regimens [2], [3]. The gain in accuracy offered by precise techniques such as stereotactic body radiotherapy (SBRT) allows higher doses to be deposited per treatment session. Prostate SBRT typically consists of delivering 7.25 Gy per fraction to the prostate, five times over two weeks, which amounts to 36.25 Gy. A recent study even demonstrated that treating with higher doses was feasible with 40 Gy to the prostate and a simultaneous boost to the Gross Tumor Volume (GTV) of 45 Gy in five fractions [4]. Such ultra-hypo-fractionated SBRT regimens were found to be as effective as conventionally fractionated radiotherapy [5], [6]. They are also associated with a substantially lower treatment cost, burden and carbon footprint [7] as they minimize treatment duration and commuting time for the patients. While there are conflicting results as to whether ultra-hypo-fractionated SBRT is associated with higher acute genito-urinary and gastro-intestinal toxicity, studies found no difference over the longer term [5], [8].

Despite being effective overall in treating prostate cancer, radiotherapy fails to control the disease for a small proportion of patients. One of the well-established causes of relapse post-radiotherapy is intra-tumoral hypoxia [9], [10]. In prostate cancer specifically, pre-radiotherapy hypoxia was found to be associated with biochemical relapse as well as local recurrence [11], [12].

Fluoromisonidazole positron emission tomography (F-MISO PET/CT) is a widely used imaging approach for visualizing tissue hypoxia. However, its use for frequent imaging is limited by its ionizing nature, the logistical requirements associated with radiotracer procurement and administration, and the time required between injection and image acquisition. Consequently, repeated imaging during a course of radiotherapy is challenging, and the daily evolution of tumor oxygenation in response to treatment remains largely unknown. In prostate cancer patients, Supiot et al. observed a decrease in hypoxia between F-MISO PET images acquired prior to radiotherapy and after the first quarter of treatment (20/79 fractions) [13], suggesting radiation-induced changes in tissue oxygenation.

Magnetic Resonance (MR) imaging has played an increasingly important role in radiotherapy [3], [14], [15], partly due to its high soft-tissue contrast and its non-ionizing nature. In recent years the UNITY Magnetic Resonance linear accelerator (MR-linac) which combines a particle accelerator ring with a 1.5 Tesla MRI was introduced into routine clinical practice. MR-linacs, primarily employed to adapt radiotherapy delivery in real time based on anatomical changes, can also be used to monitor physiological processes such as perfusion, diffusion, or tumor oxygenation in a non-invasive manner. Indeed, MRI biomarkers can be obtained during treatment, and provide insight into tumor biology potentially allowing the online monitoring of treatment response.

One such variable of interest is the relative Oxygen Extraction Fraction (rOEF), which represents the proportion of oxygen extracted from the blood by tissue. As rOEF reflects the balance between oxygen delivery and oxygen consumption, it has been proposed as an imaging biomarker related to tissue oxygenation and potentially tissue hypoxia. Different methods exist to estimate the rOEF. Previous studies using a contrast-enhanced MRI approach to estimate the rOEF have reported associations between rOEF and higher tumor grade and unfavorable molecular alterations in patients treated for a glioma [16], [17].

Alternatively, rOEF can be calculated using a combination of three non-contrast-enhanced MRI sequences. In this approach, the intravoxel incoherent motion (IVIM)-derived perfusion fraction is used as a surrogate of the vascular component. It is non-invasive and non-ionizing, does not require contrast media, and can therefore be repeated frequently. A previous study demonstrated the feasibility of acquiring rOEF maps in the healthy prostate on a UNITY MR-linac using this IVIM-based approach [18].

The purpose of the present study is to measure and map daily rOEF spatial distribution over time during SBRT for prostate cancer to provide insight on the evolution of tissue oxygenation in response to radiotherapy. This may have implications for selecting patients for treatment personalization.

## 2 Materials and methods

### 2.1 Patient cohort, MRI acquisition and rOEF calculation

A cohort of 23 prostate cancer patients treated between March 2024 and June 2025 with SBRT on the UNITY 1.5 Tesla MR-linac was selected for analysis. The prescribed doses were 36.25 Gy to the prostate and 40 Gy to the GTV, delivered in five fractions. The institutional review board approved this study (NCT06041555) and written informed consent was obtained from the participants.

At the treatment planning stage (fraction 0), as well as at successive sessions of treatment (fractions 1 to 5), three MR sequences were acquired for all patients on the UNITY MR-linac: T2-map, Multiple gradient-echo and IVIM [19], [20], [21]. The parameters used to acquire the MR sequences are provided in Supplementary Material A. By using variables extracted from these sequences, and based on the equations detailed in Supplementary Material B [18], [22], [23], 3D rOEF distributions (voxel size: 1.23 × 1.23 × 5.5 mm^3^) were calculated on the AWESOMME platform [24] for the region of interest centered on the prostate. High rOEF values reflect increased oxygen extraction. Such values are of particular interest as they may arise from reduced oxygen delivery, increased oxygen demand, or a combination of both which could be characteristics of hypoxic regions. However, the potential quantitative relationship between rOEF and tissue hypoxia has not yet been established, and rOEF should therefore be considered as an imaging biomarker related to oxygen extraction rather than a measure of hypoxia. Voxels composed of adipocytes were excluded from analysis, based on Proton Density Fat Fraction (PDFF), as they render the calculations unreliable.

Patients with rOEF images available and exploitable at less than four sessions between fraction 0 and fraction 5 were excluded. Incomplete imaging data were mainly attributable to clinical workflow limitations such as delayed or postponed treatments at the MR-linac.

### 2.2 Image registration and rOEF extraction

Prostate and GTV contours from the clinical treatment plan were first transferred to T2 images using rigid registration based on prostate tissue. Then the delineations were copied to the rOEF images where they were used to extract rOEF data in the regions of interest. All GTV contours were validated by a radiation oncologist, expert in prostate cancer radiotherapy.

Average rOEF values were extracted at every available fraction for the healthy prostate (prostate excluding GTV), and the GTV.

To investigate whether changes in rOEF during treatment were associated with radiation exposure, rOEF in gluteal muscle was also extracted in two anatomical regions: in-field muscle exposed to residual irradiation and out-of-field muscle exposed to negligible dose, as detailed in Supplementary Material C. The out-of-field muscle, which received negligible dose compared with the other regions studied, was used as a reference for comparison.

Also, the proportion of GTV voxels exceeding predefined rOEF thresholds of >30%, >50% and > 70%, named hereafter high-rOEF fraction, was calculated. Threshold values were chosen to cover a broad spectrum as the literature currently lacks information on a potential rOEF-hypoxia relationship. For patients presenting with multiple malignant lesions, high-rOEF fractions were calculated by pooling the volumes of the lesions together into one variable per patient.

### 2.3 Longitudinal analysis of rOEF changes

Changes over time of average rOEF and GTV high-rOEF fractions were quantified by linearly fitting the data. Slopes of the linear models were extracted to determine if there is an overall trend over time. *t*-tests were then used to assess whether the distributions of the 23 slopes extracted (one per patient) for each variable were statistically significantly (*p*-value ≤0.05) differing from 0. As such, a statistically significant result with a negative median of slopes would indicate a decrease over time of the analyzed variable.

Image and data analyses were performed using MatLab version R2022a.

### 2.4 Robustness to contouring uncertainty

The robustness of rOEF trends to GTV contouring uncertainties was assessed. To do so, four margins of two voxels were alternatively added to the GTV contours, in the anterior, posterior, right and left directions (Supplementary Material D). To quantify the impact of these variations in GTV delineation on rOEF variables, slopes were extracted from the four scenarios and their distributions tested using a *t*-test as described above.

### 2.5 Relationship between tumor volume and elevated rOEF

To assess whether larger tumors are more likely to contain elevated rOEF regions, high-rOEF fractions were quantified at fraction 0 and plotted as a function of total GTV volumes. Patients with multiple GTVs were excluded from this analysis.

### 2.6 Comparison of rOEF between tumor and healthy prostate

To determine whether rOEF differed between the GTV and healthy prostate tissue and whether this difference changed over time, mean rOEF values were calculated and plotted by treatment fraction. Paired-sample *t*-tests were used to assess if the distributions of average rOEF for GTV and prostatic tissue could be considered statistically significantly different.

### 2.7 Temporal evolution of prostate rOEF

For this analysis, only fractions for which irradiation occurred before MRI acquisition were included. For fraction 1, the irradiation-MRI sequence for all patients is provided in Supplementary Material E. For the average rOEF of the healthy prostate, five slopes were calculated between successive fractions (0–1, 1–2, through 4–5), depending on data availability. To visualize where the maximum rOEF variations occur during SBRT, median slope values across patients were then plotted as a function of fraction number.

## 3 Results

Out of the 23 patients analyzed, eight had a complete dataset with all six rOEF images (from fraction 0 to fraction 5), ten had one missing rOEF image and five had two. The missing data are distributed as detailed in Supplementary Material F.

An illustration of the evolution during SBRT of 3D rOEF distributions can be seen in Fig. 1 for two patients for slices including GTV contours. While some high rOEF regions appear stable from fraction to fraction, others are volatile. Also, visually it appears that the overall rOEF decreases during the whole SBRT course.

**Fig. 1:**
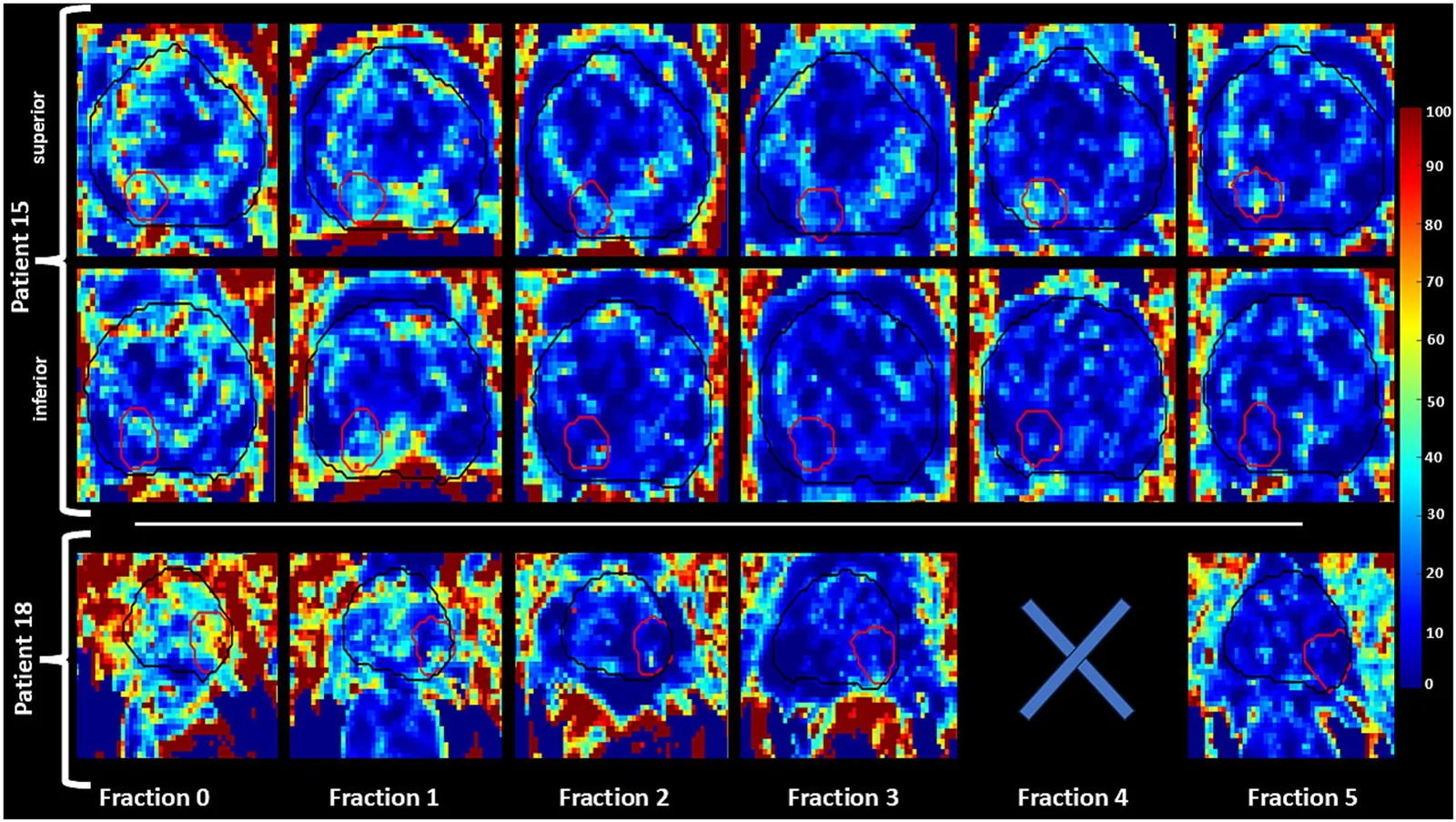
Visualization for two patients (number 15 and 18) of rOEF maps during SBRT with prostate and GTV delineations in black and red, respectively. (For interpretation of the references to colour in this figure legend, the reader is referred to the web version of this article.)

Average rOEF values for the healthy prostate and for the GTV are shown in Fig. 2 Panels A and B. For the healthy prostate, the median (range) of the average rOEF across patients decreased from 24% (14%–55%) at fraction 0 to 13% (8%–22%) at fraction 5. For the GTV, the median (range) rOEF also declined between fraction 0 and 5 from 19% (2%–52%) to 15% (7%–40%). Median (range) high rOEF fractions of the GTV were found to drop from 3% (0%–49%) to 1% (0%–25%) between fraction 0 and 5 for the >50% threshold and from 2% (0%–18%) to 0% (0%–9%) for the >70% threshold (Fig. 2 Panels C and D).

**Fig. 2:**
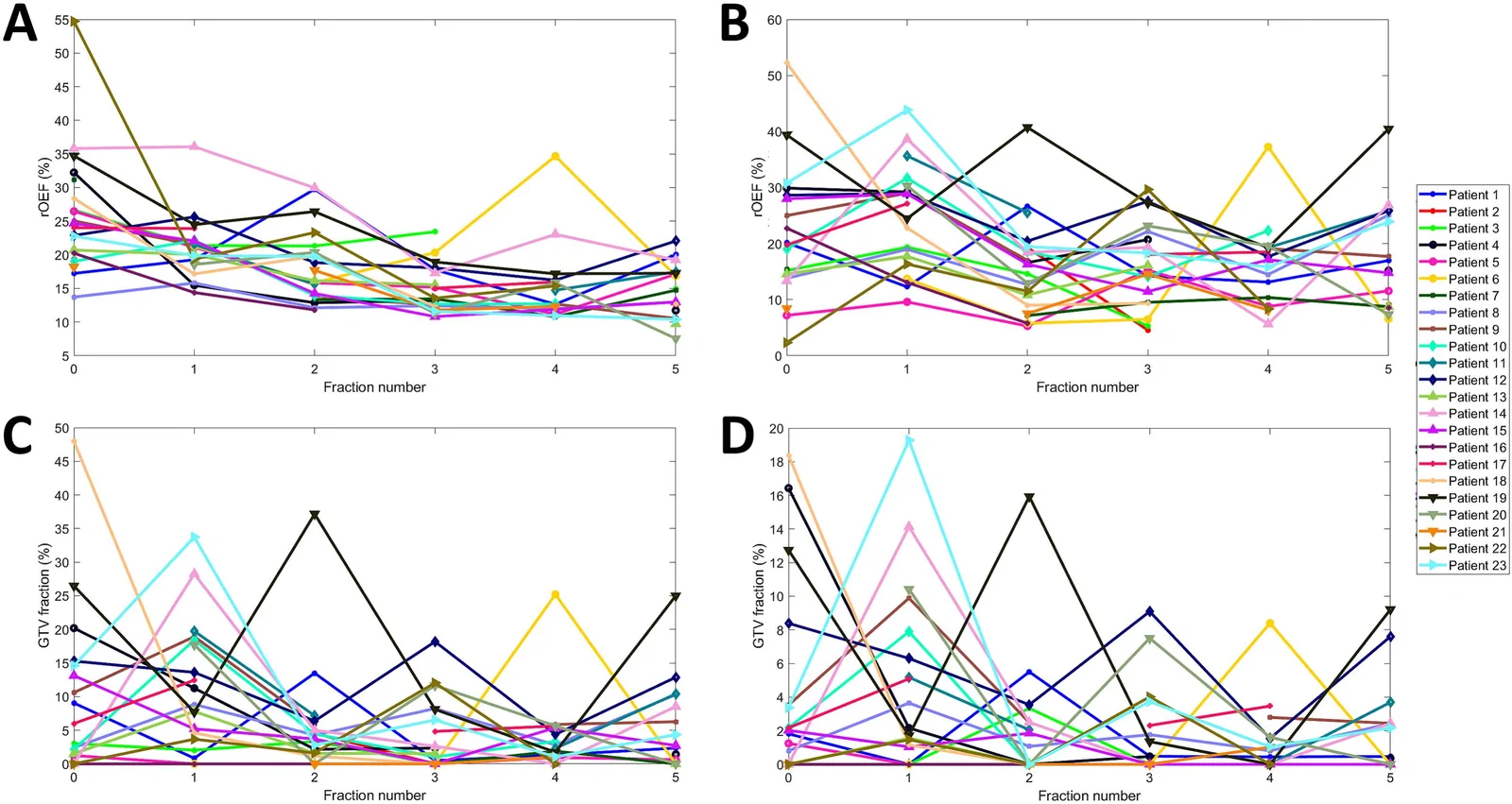
Panel A and Panel B show average rOEF values over time for the healthy prostate and the GTV, respectively. Panel C and Panel D show the high-rOEF fraction of the GTV over time as defined by two thresholds: rOEF >50% and > 70%, respectively.

Average rOEF slopes were significantly negative in all irradiated regions (healthy prostate, GTV, and in-field gluteal muscle), indicating an overall decrease in average rOEF during SBRT (Fig. 3). In contrast, average rOEF slopes in the out-of-field gluteal muscle did not differ significantly from zero (*p* > 0.3), indicating no evidence of a systematic change during SBRT. The three GTV high-rOEF fractions studied were also found to decrease over time with statistically significant results (p < 0.01) confirming that regions exhibiting elevated rOEF values (e.g. >70%) within the tumor also decrease over the course of treatment.

**Fig. 3:**
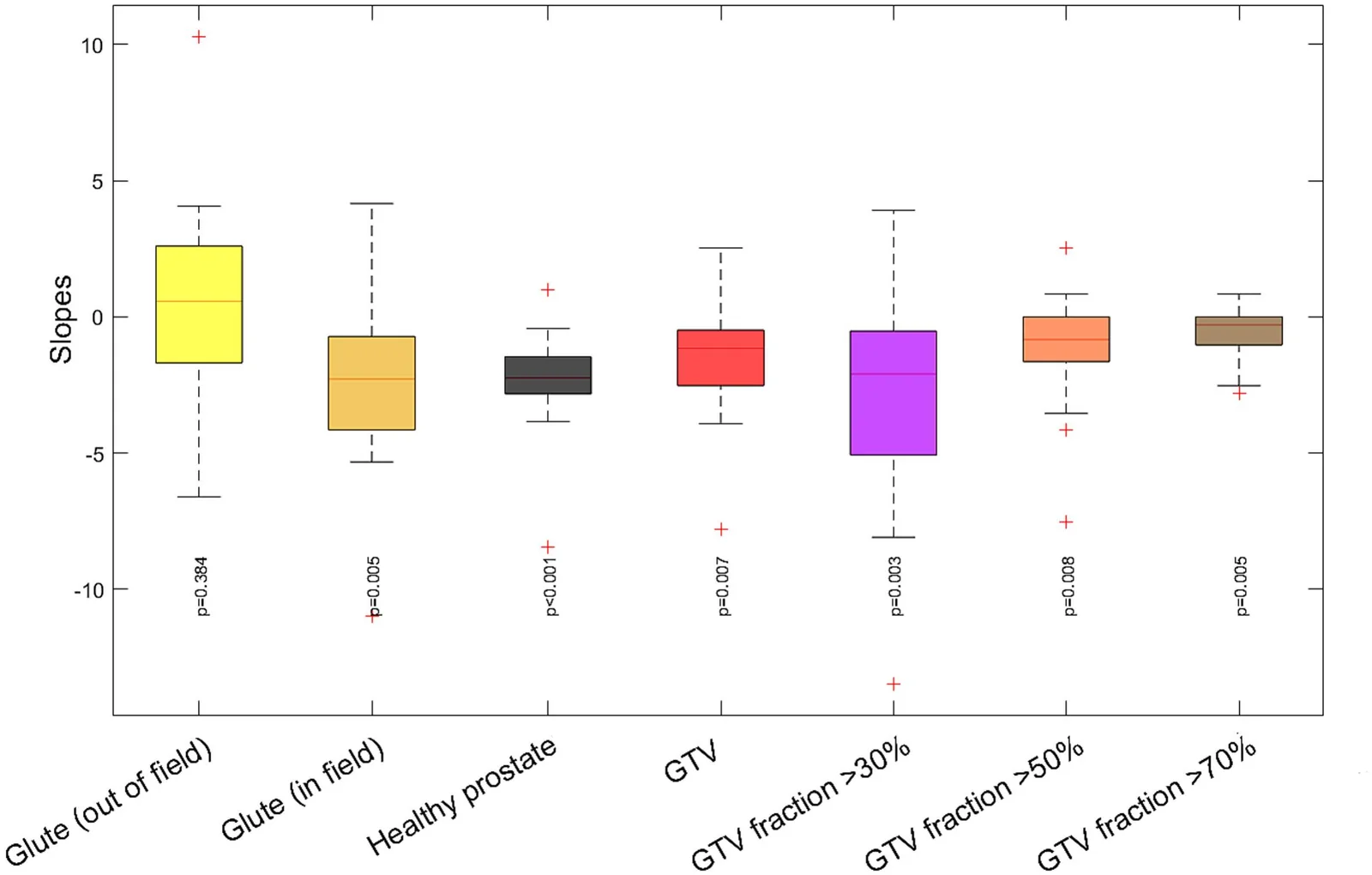
Boxplots showing the distribution of slopes in average rOEF (for out-of-field and in-field gluteal muscle, healthy prostate and GTV) as well as slopes of GTV high-rOEF fractions for three threshold values (rOEF>30%, >50% and > 70%).

Addition of uncertainty margins to the GTV resulted in median differences [Q1, Q3] in average rOEF of −0.2 [−1.4;1], −0.2 [−1.9;0.9], 0.3 [−1.2;1.3] and 0.2 [−0.9;1.2] for GTVs enlarged with an anterior, posterior, right and left margin, respectively (Supplementary Material G).

The decrease observed for the GTV in average rOEF as well as in the high-rOEF fractions is robust to contouring uncertainties (Supplementary Material H). Specifically, in all scenarios tested, the distributions of slopes remain statistically significantly negative (p < 0.05) confirming the robustness of the trends observed with unaltered delineations.

All four tumors smaller than 0.75 cm^3^ have <3% of their volumes with rOEF values >50% (Fig. 4). For larger tumors, however, regions with rOEF values >50% can cover up to 25% of their volume. Nevertheless, larger tumors are not systematically more prone to larger proportions with high rOEF as a tumor of 2 cm^3^ had only 2% of its volume with rOEF exceeding 50%.

**Fig. 4:**
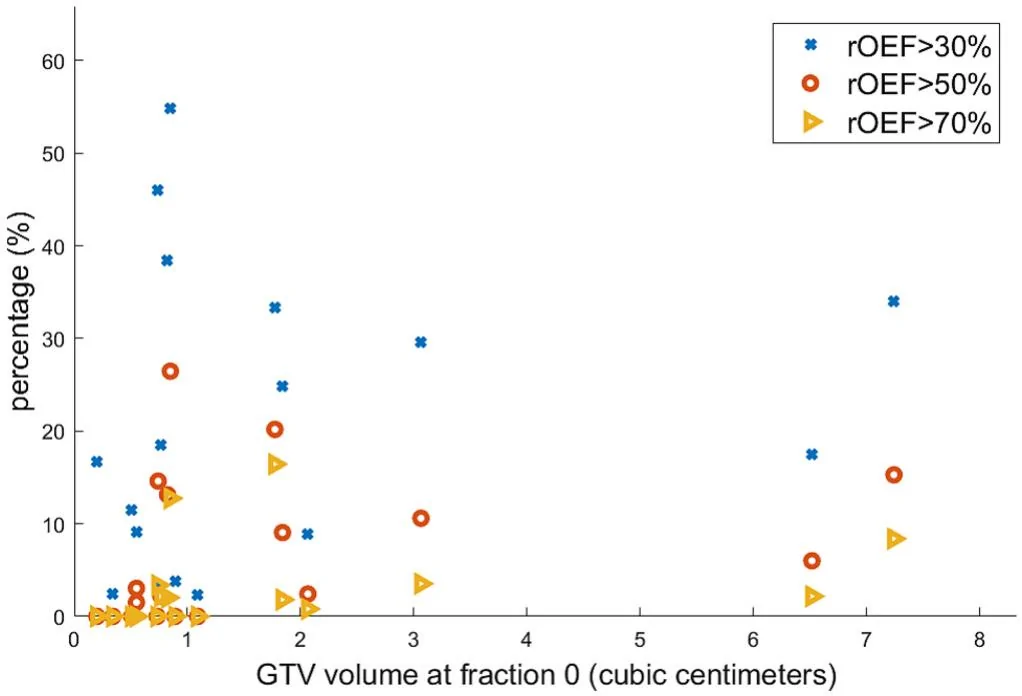
High-rOEF fractions of the GTV for three threshold values (rOEF >30%, >50% and > 70%) as a function of GTV volume at fraction 0.

Although median rOEF was higher in the GTV than in the healthy prostate at fractions 1, 3, 4 and 5, none of these differences was statistically significant (Fig. 5).

**Fig. 5:**
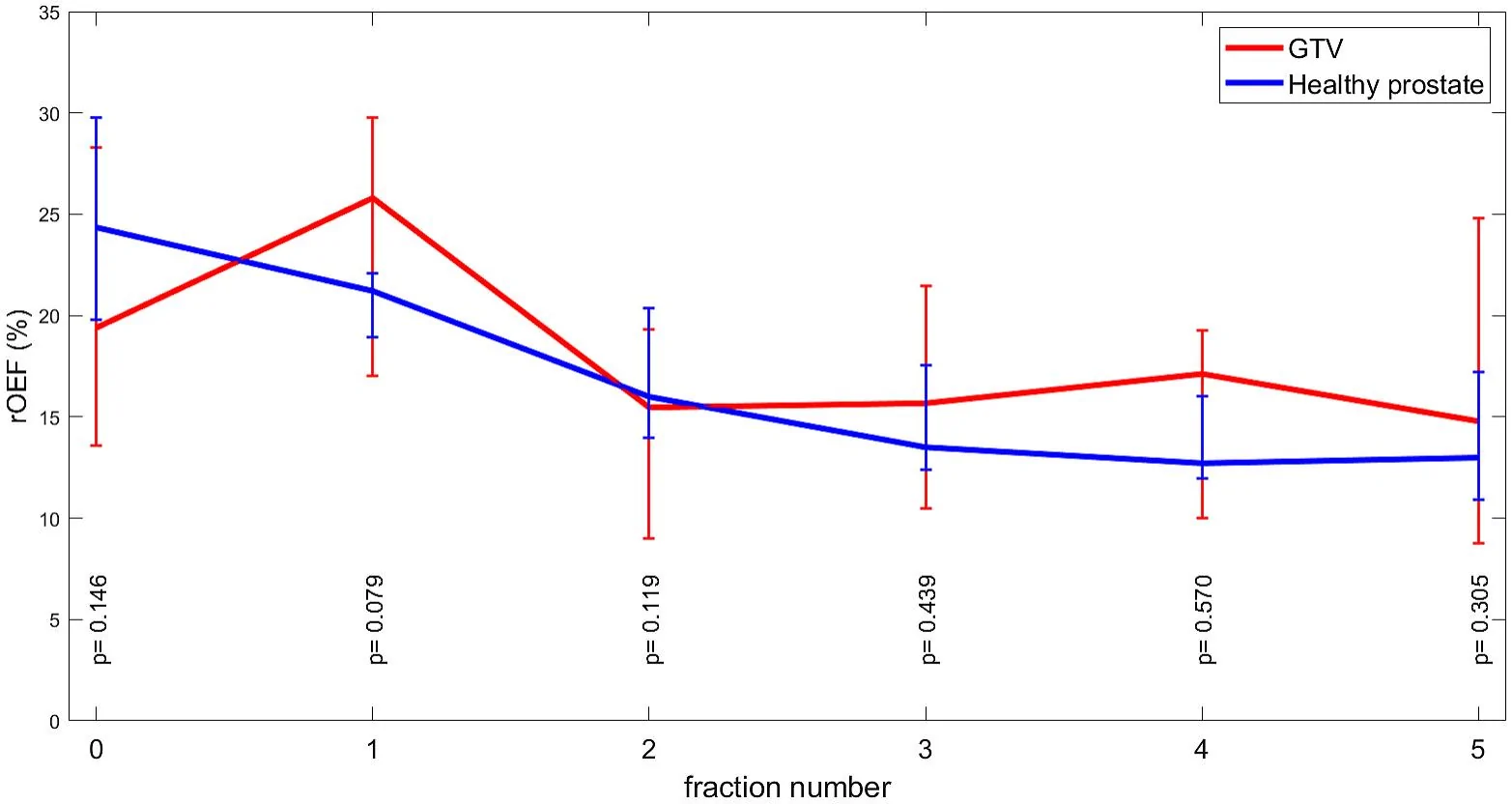
Median and first and third quartiles of average rOEF values across patients for the GTV and healthy prostate at each fraction.

As shown in Fig. 6, among patients for whom the fraction-1 MRI was acquired after the first irradiation, the median slope in healthy prostate rOEF was steepest between fractions 0 and 1, at −3.2. It remained nearly as steep between fraction 1 and 2 with −3.1. The median slopes then became less steep with a value of −1.3 for fractions 2–3 and then became even shallower with −1.2 and 0.5 for fractions 3–4 and 4–5.

**Fig. 6:**
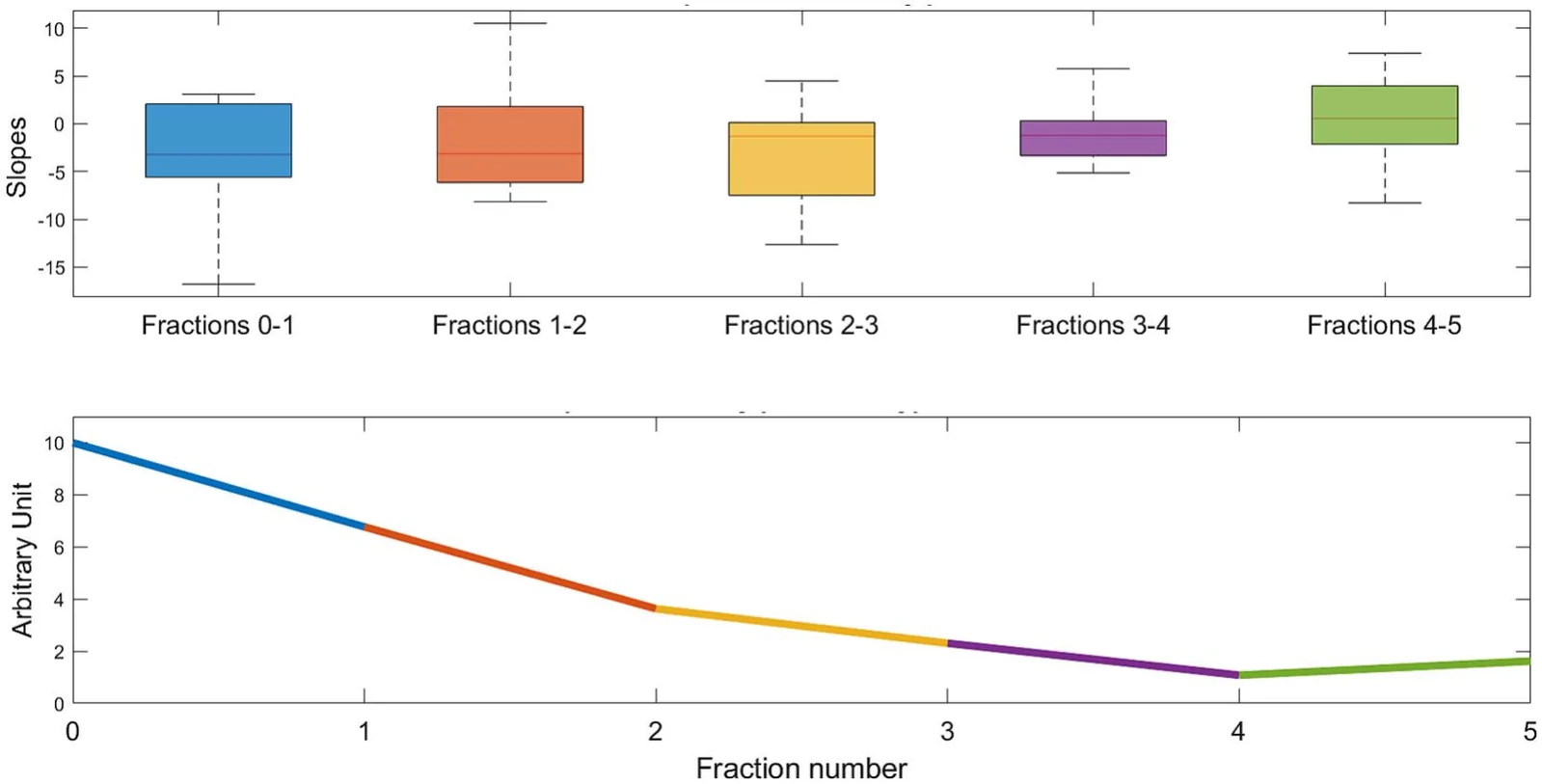
The boxplots show distributions of slopes between successive fractions (fraction 0 and 1, fraction 1 and 2… until fraction 4 and 5). The panel at the bottom shows the median values of the boxplots to visualize the evolution over time.

## 4 Discussion

The analysis of rOEF variations in response to prostate SBRT demonstrated an overall decrease in average rOEF in the irradiated regions, as well as in GTV high-rOEF fractions, whereas no systematic change in rOEF was observed in the minimally irradiated out-of-field gluteal muscle. This pattern is consistent with an association between rOEF changes and radiation exposure.

Our results are broadly consistent with those of previous studies that reported changes in tumor oxygenation during radiotherapy in prostate, lung and head and neck cancer, using commonly used hypoxia imaging techniques [13], [25]. However, these previous studies assessed hypoxia-related imaging biomarkers at two to three time points during treatment as they used ionizing methods, which limits the sampling frequency. A strength of the present study is the daily availability of MR-images that allowed us to quantify the kinetics of rOEF. We found that, on average, rOEF of the healthy prostate decreased during the first four fractions before plateauing at the end of SBRT (Fig. 6). This temporal pattern is of interest in the context of previous studies reporting tumor re‑oxygenation during the early phase of radiotherapy [26], although the present data do not establish that the observed decrease in rOEF represent radiobiological re‑oxygenation.

In the present study, rOEF was analyzed as an MRI-derived biomarker related to tissue oxygenation rather than as a direct measure of tissue hypoxia. Physiologically, rOEF reflects the balance between oxygen delivery and oxygen consumption. Consequently, elevated rOEF values may arise from reduced oxygen delivery, increased metabolic demand, or a combination of both. Likewise, the reduction in rOEF observed during SBRT may be consistent with increased oxygen availability, potentially due to increased tissue perfusion or changes in vascular function, reduced oxygen consumption following irradiation (e.g. due to cell inactivation), or multiple concurrent physiological processes. Although previous studies using hypoxia imaging approaches have reported reoxygenation during radiotherapy [13], [25], our findings may be consistent with an increase in tissue oxygen availability but do not establish whether this represents classical radiobiological reoxygenation or another physiological process.

The methodology used to derive rOEF also differs from that used in some previous studies. An alternative approach to estimating rOEF, reported by Oughourlian et al. [17], uses R2’ and Dynamic Susceptibility Contrast-derived relative cerebral blood volume. Although this approach shares a similar physical basis with the present IVIM-based method, which uses the perfusion fraction as the vascular component, the resulting rOEF estimates cannot currently be considered directly comparable or interchangeable, in the absence of a direct comparison between the two methods.

It is particularly interesting to note that the average rOEF of the healthy prostate decreased between fractions 0 and 1 (Fig. 6) in the subset of patients for whom the fraction-1 MRI was acquired after the first irradiation. Thus, the decrease was detectable on the MRI acquired after the first irradiation, suggesting that radiation-associated physiological changes may occur during this early post-treatment period. However, the present data cannot determine when during the interval between the two MRI acquisitions the change occurred. Further studies will be required to characterise the temporal dynamics and determine the biological mechanisms underlying these changes.

Interestingly, Fig. 1 also illustrates regions of elevated rOEF surrounding the prostate at the beginning of treatment, which appear to decrease progressively over the course of SBRT. Given their close proximity to the prostate, these regions may have received appreciable radiation exposure, consistent with the observed decrease in rOEF in other irradiated tissues. Although the clinical significance of these findings cannot be established from the present data alone, this observation further supports an association between rOEF changes and radiation exposure.

In the present study, patients with four or less fractions available were excluded from analysis. In the selected cohort, ten patients had one missing rOEF image and five had two (Supplementary Material F). Although these missing fractions may have influenced the estimated temporal trends, the observed decreases in the irradiated regions remained statistically significant.

The fact that delineations made on T2 MRI with high resolution were transferred to images with lower spatial resolution potentially represents another source of uncertainty. A consequence of potential mismatch in slice thickness is that GTV volumes varied from fraction to fraction (Supplementary Material I). These variations led us to calculate the high-rOEF fractions (proportion of the GTV above a certain rOEF threshold) instead of extracting only absolute volumes as this approach is robust to these variations. More broadly, sensitivity analyses using expanded GTV contours showed that the observed rOEF trends were robust to these contouring uncertainties (Supplementary Materials H).

Furthermore, the potential relationship between rOEF and tumor hypoxia has not yet been established. Therefore, we used three threshold values (rOEF>30%,>50% and > 70%) for defining the high-rOEF fractions. A future study will investigate this relationship by comparing FMISO-PET images with rOEF, which may help identify an rOEF threshold associated with hypoxia.

Having access to daily maps of hypoxia could enable a shift from purely geometry-based adaptation, to biologically guided radiotherapy, potentially improving personalization and outcomes. If the relationship between rOEF and tumor hypoxia is established, such a dose-painting scenario could consist of targeting regions with elevated rOEF, as identified just prior to irradiation on the MR-linac, with a dose boost. The observed changes in the spatial distribution of regions with elevated rOEF suggest that, if rOEF is validated as a marker of tumor hypoxia, these regions could be boosted at each fraction based on daily rOEF mapping rather than relying on a single baseline map. Such an approach would be enabled by MR imaging, which can provide daily 3D maps without additional radiation exposure.

In conclusion, rOEF progressively decreased in irradiated regions during prostate SBRT, while no significant change was observed in minimally-irradiated tissue. These findings suggest an association between radiation exposure and changes in physiological processes influencing tissue oxygen extraction. The decrease in healthy prostate rOEF was detectable after the first irradiation, although the temporal dynamics and underlying biological mechanisms remain to be established. Future studies should investigate whether rOEF could provide information relevant to tumor radioresistance and support biologically adaptive radiotherapy.

## Declaration of generative AI in the manuscript preparation process

During the preparation of this work, the authors used ChatGPT v5 to improve the grammar, readability, flow, and clarity of the manuscript. After using this tool, the authors reviewed and edited the content as needed and take full responsibility for the content of the published article.

## CRediT authorship contribution statement

**Thomas Berger:** Writing – original draft, Visualization, Software, Methodology, Investigation, Formal analysis, Data curation, Conceptualization. **Ariane Lapierre:** Writing – review & editing. **Perla Zidane:** Writing – review & editing, Methodology. **Hayder Aloraibi:** Writing – review & editing, Methodology. **Samy Horn:** Writing – review & editing, Resources. **Angela Boros:** Writing – review & editing, Methodology. **Frederic Cervenansky:** Writing – review & editing, Software, Data curation. **Benjamin Leporq:** Writing – review & editing, Software, Methodology, Formal analysis, Data curation. **Olivier Beuf:** Writing – review & editing, Supervision, Software, Methodology, Data curation, Conceptualization. **William Henry Nailon:** Writing – review & editing, Supervision, Methodology. **Olivier Chapet:** Writing – review & editing, Supervision, Resources, Project administration, Methodology, Funding acquisition, Conceptualization.

## Declaration of competing interest

Given their role as Associate Editor, William Nailon had no involvement in the peer-review of this article and has no access to information regarding its peer-review. Full responsibility for the editorial process for this article was delegated to another journal editor.

The authors declare the following financial interests/personal relationships which may be considered as potential competing interests: Thomas Berger, Perla Zidane and Olivier Chapet report financial support was provided by Elekta. Olivier Chapet reports a relationship with Elekta that includes: unrestricted funding grants. Olivier Beuf, Frederic Cervenansky and Benjamin Leporq report financial support was provided by LabEx PRIMES. If there are other authors, they declare that they have no known competing financial interests or personal relationships that could have appeared to influence the work reported in this paper.

## Data Availability

The data analyzed in this study can be shared upon reasonable request to the corresponding author, subject to institutional and patient confidentiality regulations.
