# Supplementary material for: 3D analysis and evolution of prostate and tumor oxygenation using MRI-derived relative oxygen extraction fraction during MR-guided stereotactic body radiotherapy
Source: Phys Imaging Radiat Oncol. 2026 Oct 1;41:101097. doi: 10.1016/j.phro.2026.101097 (PMC13660655; doi:10.1016/j.phro.2026.101097)

Supplementary Materials

Supplementary Material A: Acquisition parameters for the MR sequences

Map	T2 mapping	T2* mapping (CS-imaging)	IVIM
Type of sequence	Multiple-echo Turbo Spin Echo	Multi-Gradient-Echo FFE	Single-shot echo planar image (ss-EPI)
b-values (average) (s/mm²)	-	-	0 (2), 10 (2), 40 (4), 80 (4), 200 (8), 500 (8)
Δ TE (ms)	15	2.4	-
TE (number of echoes) (ms)	15-90 (6)	4.6-21.8 (8)	68
TR (ms)	11800	5577	44645

Supplementary Material B: Procedure for quantitative MRI parameters calculation

T2 mapping

T2 maps were calculated fitting the eq.1 to the data using a constrained non-linear least square fit:

$$S_n = S_0 e^{-\frac{TE_n}{T2}} \quad \text{(Eq.1)}$$

where S_n is the signal value at TE_n, and S_0 is the signal value at $TE_n = 0$. To avoid T2 overestimation, T2 was calculated by discarding the first echo.

IVIM parameters mapping

The IVIM parameters were calculated by fitting the Eq.2 to the multiple b-values images using an optimization algorithm based on a constrained Bayesian-inference method.

$$S_b = S_0\left(f e^{-b.D_{fast}} + (1-f)e^{-b.D_{slow}}\right) \quad \text{(Eq.2)}$$

where D_{fast} is the pseudo-diffusion coefficient, f is the perfusion fraction, D_{slow} is the true diffusion coefficient, and S_0 is the signal value at b = 0.

Calculation of blood volume (BV):

The IVIM perfusion fraction (f) was first corrected by the blood-tissue T2 difference according to:

$$\text{f\_corr} = \underset{x}{\text{argmin}} \frac{x.*\exp^{-TE_{dwi}\cdot/T2_{blood}}}{x.*\exp^{-TE_{dwi}\cdot/T2_{blood}} + (1-x).*\exp^{-TE_{dwi}\cdot/T2(i,j)}} - f(i,j) \quad \text{(Eq.3)}$$

With TE_{dwi} the echo time of the diffusion sequence, $T2(i,j)$ and $f(i,j)$the voxel-wise measured T2 and f. Blood R2 (1/T2blood) was fixed according to the quadratic relation linked the mixed blood O2 saturation (Y) for a given hematocrit, at 1.5T:

$$R2_{blood} = A + B.(1-Y) + C.(1-Y)^2 \quad \text{(Eq.4)}$$

Y was assumed equal to 0.75. A, B and C are fitted coefficients determined experimentally according to (Silvennoinen M.J et al. Magn Reson Med. 2003;49:47-60)

Then, the corrected perfusion fraction (f_corr) was normalized by the fat fraction to obtain the blood volume (BV):

$$BV = (1 - PDFF) * \text{f\_corr} \quad \text{(Eq.5)}$$

T2* and PDFF and mapping

To avoid chemical shift effect, T2* mapping was done simultaneously with fat-water decomposition using the method described in (Leporq B, et al. NMR Biomed. 2014 Oct;27(10):1211-21. doi: 10.1002/nbm.3175). Briefly, a phase correction algorithm was used to unwrap and correct the native phase images for zero- and first-order phase and rebuild the B0-demodulated real part images. Then, using a model of fat 1H MR spectrum integrating eight components, T2* and PDFF was estimated voxel by voxel by from a constrained non-linear least square fit on the real part of the corrected signal.

Multi-parametric oxygenation mapping

The reversible transverse relaxation coefficient $R2' = {}^{1}/_{T2'}$, corresponding to the measure of mesoscopic field inhomogeneities variation assumed to be linked to the susceptibility effect on MR signal induced by variation of blood level of oxyhemoglobin and deoxyhemoglobin. This can be written mathematically as the following equation:

$$R2' = R2^* - R2 \quad \text{(Eq.6)}$$

where $R2^* = {}^{1}/_{T2^*}$ and $R2 = {}^{1}/_{T2}$ are respectively derived from T2 and T2* maps.

R2' is related to field contribution (c) induced by the difference between the magnetic susceptibilities of fully oxygenated and fully deoxygenated hemoglobin ($\Delta\chi$), the tissue consumption in oxygen (relative oxygen extraction fraction = rOEF), the blood volume (BV) in the microcapillary compartment such as:

$$rOEF = \frac{R_2'}{BV \times c} \quad \text{(Eq.7)} \quad \text{and} \quad c = \frac{4}{3}\pi\gamma\Delta\chi Ht B_0 \quad \text{(Eq.8)}$$

with $\Delta\chi$ the magnetic susceptibility differences between oxy- and desoxyhemoglobin (0.264 ×10-6 CGS), B0 the magnetic static field intensity (1.5T) and γ the 1H gyromagnetic ratio (2.67502 ×108 rad/s/T) and Ht the hematocrit of small vessel considered at 0.42 x 0.75.

Supplementary Material C : Illustration for patient 3 of in-field and out-of-field gluteal muscle selections.

The in-field gluteal muscle shown in the rOEF image just below in yellow was selected on four slices within the cranio-caudal extension of the prostate (here in black). Given the proximity between the target and the in-field gluteal muscle, it is exposed to residual irradiation.

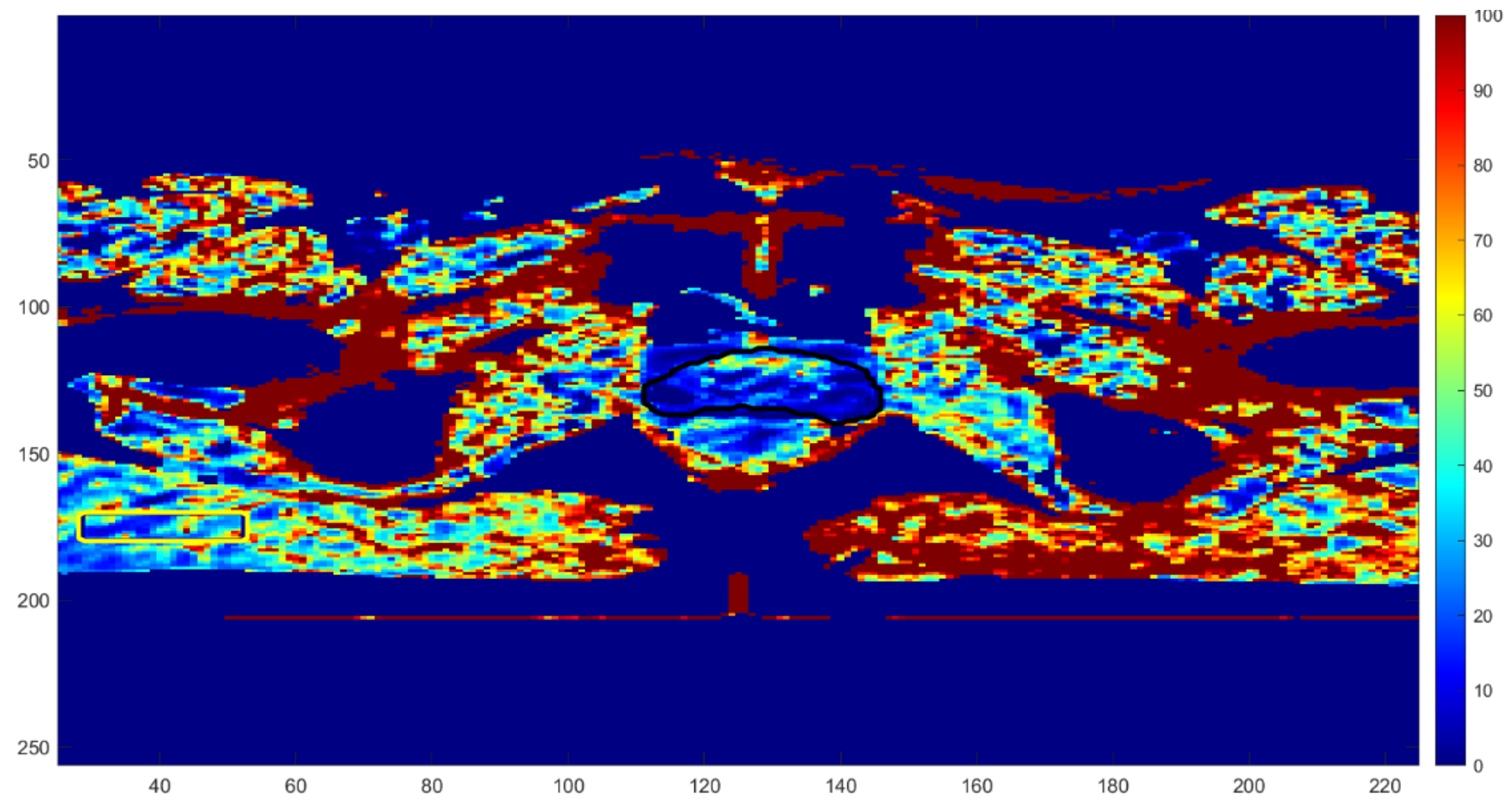

The out-of-field gluteal muscle, shown in the rOEF image just below in yellow, was selected over three slices at least one slice above the prostate i.e. at the bladder level where insignificant levels of radiation are deposited.

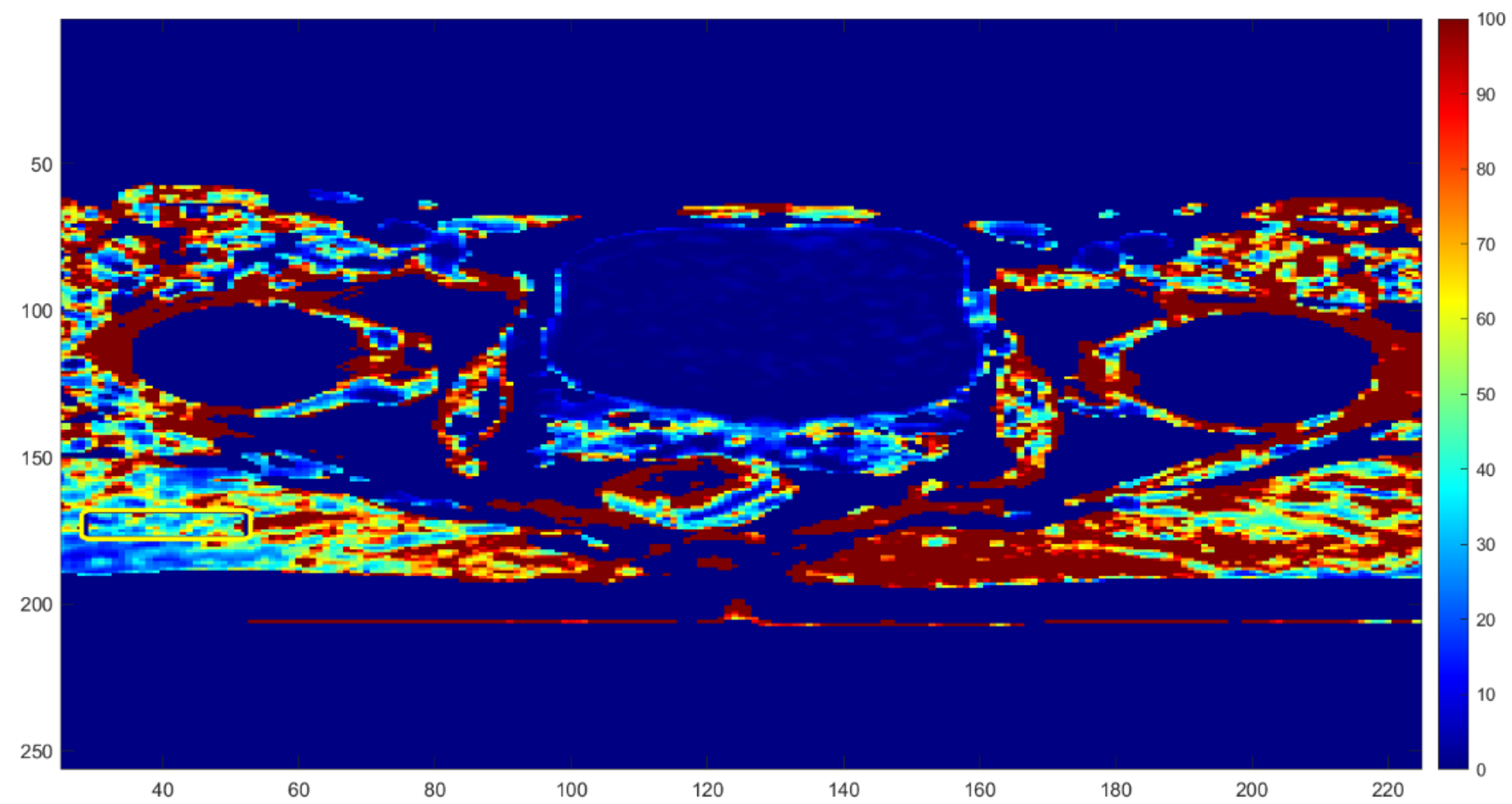

Supplementary Material D : Illustration for one patient (patient 4) on an rOEF map, of the two-voxel expansion of GTV delineations (in red) used to assess the findings' robustness to contouring uncertainties. GTV delineations were expanded in four directions: right (magenta), left (sky blue), anteriorly (green) and posteriorly (yellow).

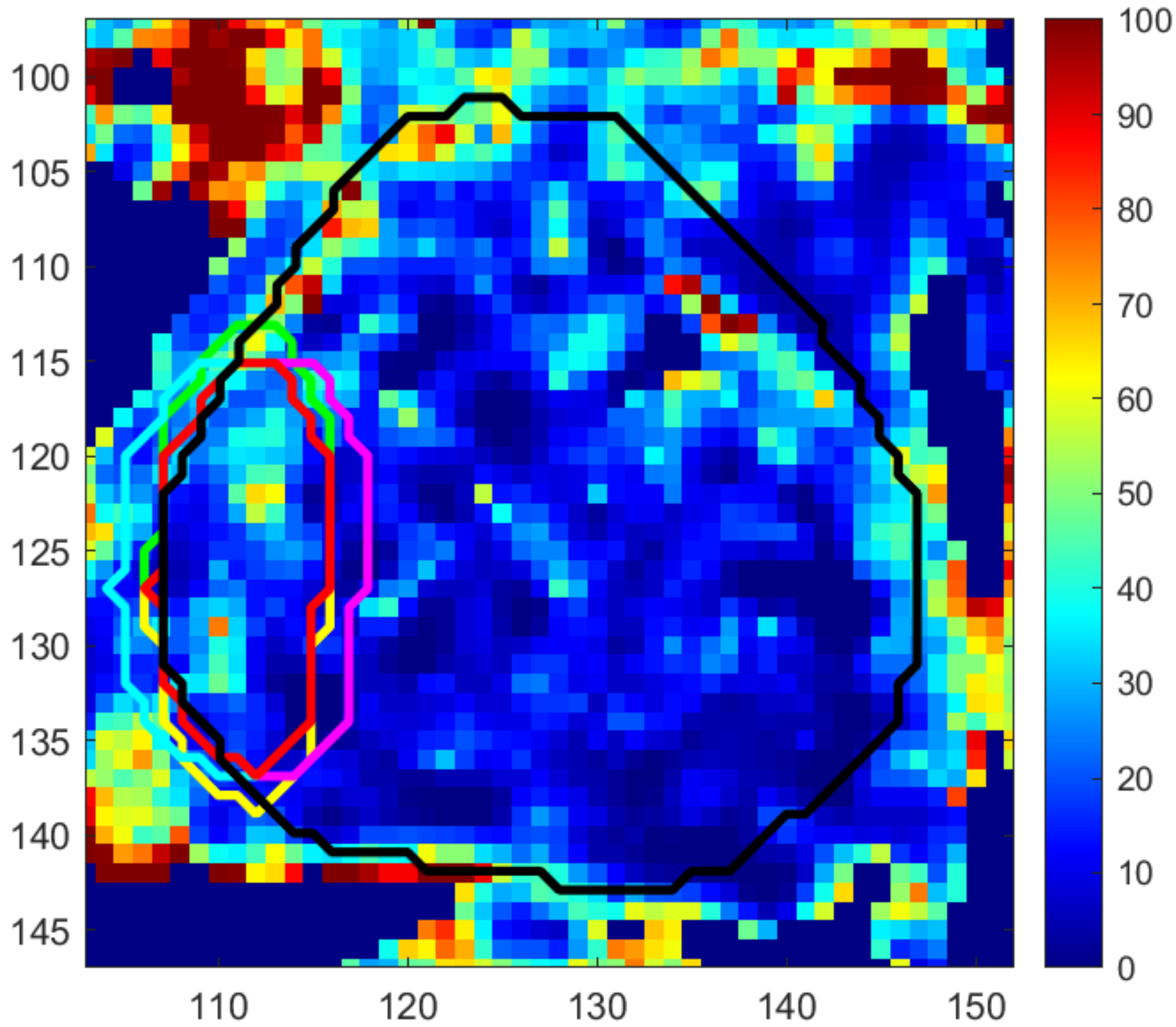

Supplementary Material E : Irradiation relative to MRI acquisition for fraction 1.

	Fraction 1
Patient 1	Irradiation preceding MRI
Patient 2	NA
Patient 3	Irradiation preceding MRI
Patient 4	Irradiation preceding MRI
Patient 5	Irradiation preceding MRI
Patient 6	Irradiation preceding MRI
Patient 7	NA
Patient 8	Irradiation preceding MRI
Patient 9	Irradiation preceding MRI
Patient 10	Irradiation preceding MRI
Patient 11	Irradiation preceding MRI
Patient 12	Irradiation preceding MRI
Patient 13	Irradiation preceding MRI
Patient 14	Irradiation not preceding MRI
Patient 15	Irradiation not preceding MRI
Patient 16	Irradiation preceding MRI
Patient 17	Irradiation not preceding MRI
Patient 18	Irradiation not preceding MRI
Patient 19	Irradiation preceding MRI
Patient 20	Irradiation preceding MRI
Patient 21	NA
Patient 22	Irradiation not preceding MRI
Patient 23	Irradiation preceding MRI

Supplementary Material F : List of exploitable rOEF images for the cohort studied.

Patient identifier	Fraction 0	Fraction 1	Fraction 2	Fraction 3	Fraction 4	Fraction 5
Patient 1	Analysed	Analysed	Analysed	Analysed	Analysed	Analysed
Patient 2	Analysed	**Not available**	Analysed	Analysed	**Not available**	Analysed
Patient 3	Analysed	Analysed	Analysed	Analysed	**Not available**	Analysed
Patient 4	Analysed	Analysed	Analysed	Analysed	**Not available**	Analysed
Patient 5	Analysed	Analysed	Analysed	Analysed	Analysed	Analysed
Patient 6	**Not available**	Analysed	Analysed	Analysed	Analysed	Analysed
Patient 7	Analysed	**Not available**	Analysed	Analysed	Analysed	Analysed
Patient 8	Analysed	Analysed	Analysed	Analysed	Analysed	Analysed
Patient 9	Analysed	Analysed	Analysed	**Not available**	Analysed	Analysed
Patient 10	Analysed	Analysed	Analysed	Analysed	Analysed	**Not available**
Patient 11	**Not available**	Analysed	Analysed	**Not available**	Analysed	Analysed
Patient 12	Analysed	Analysed	Analysed	Analysed	Analysed	Analysed
Patient 13	Analysed	Analysed	Analysed	Analysed	**Not available**	Analysed
Patient 14	Analysed	Analysed	Analysed	Analysed	Analysed	Analysed
Patient 15	Analysed	Analysed	Analysed	Analysed	Analysed	Analysed
Patient 16	Analysed	Analysed	Analysed	**Not available**	**Not available**	Analysed
Patient 17	Analysed	Analysed	**Not available**	Analysed	Analysed	**Not available**
Patient 18	Analysed	Analysed	Analysed	Analysed	**Not available**	Analysed
Patient 19	Analysed	Analysed	Analysed	Analysed	Analysed	Analysed
Patient 20	**Not available**	Analysed	Analysed	Analysed	Analysed	Analysed
Patient 21	Analysed	**Not available**	Analysed	Analysed	Analysed	**Not available**
Patient 22	Analysed	Analysed	Analysed	Analysed	Analysed	**Not available**
Patient 23	Analysed	Analysed	Analysed	Analysed	Analysed	Analysed

Supplementary Material G : Boxplot showing absolute difference in average rOEF between GTV and GTV with contouring uncertainties in the four scenarios tested (anterior, posterior, right and left margin of two voxels).

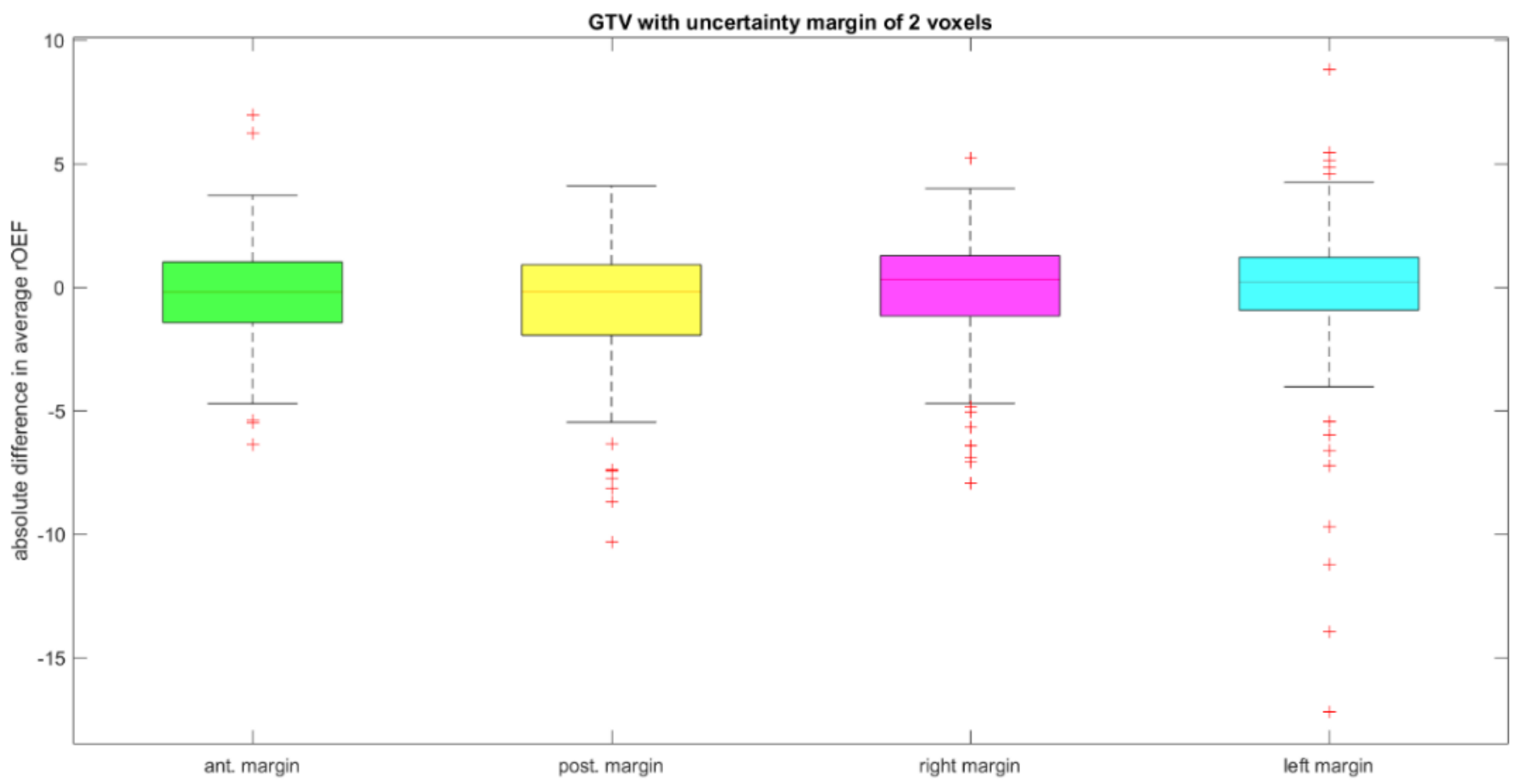

Supplementary Material H : Boxplot showing slopes across patients for average rOEF in the GTV and for the GTV high rOEF fractions for the four scenarios with contouring uncertainties (anterior, posterior, right and left margin of two voxels).

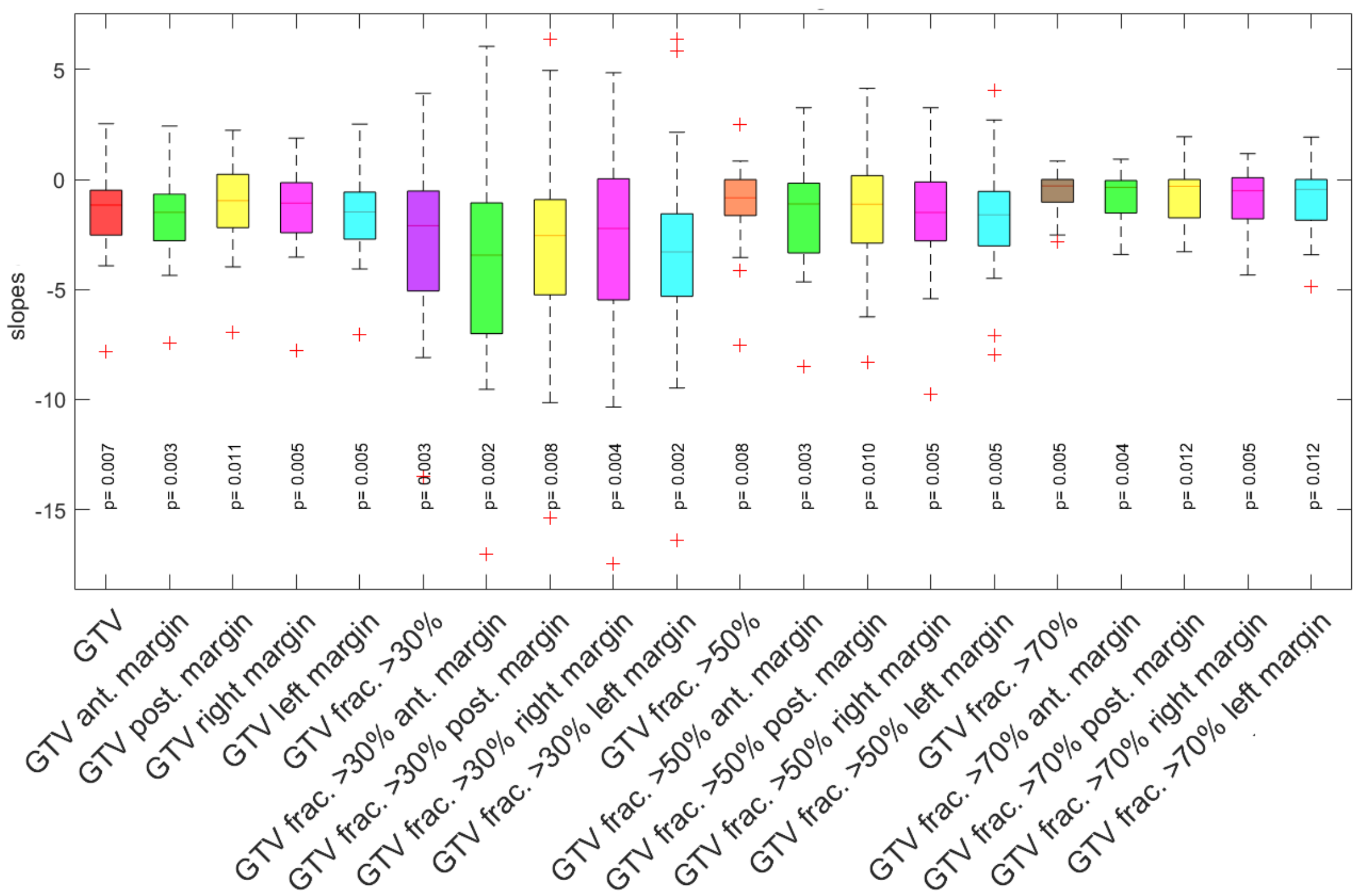

Supplementary Material I : GTV volume as a function of fraction number for the cohort studied.

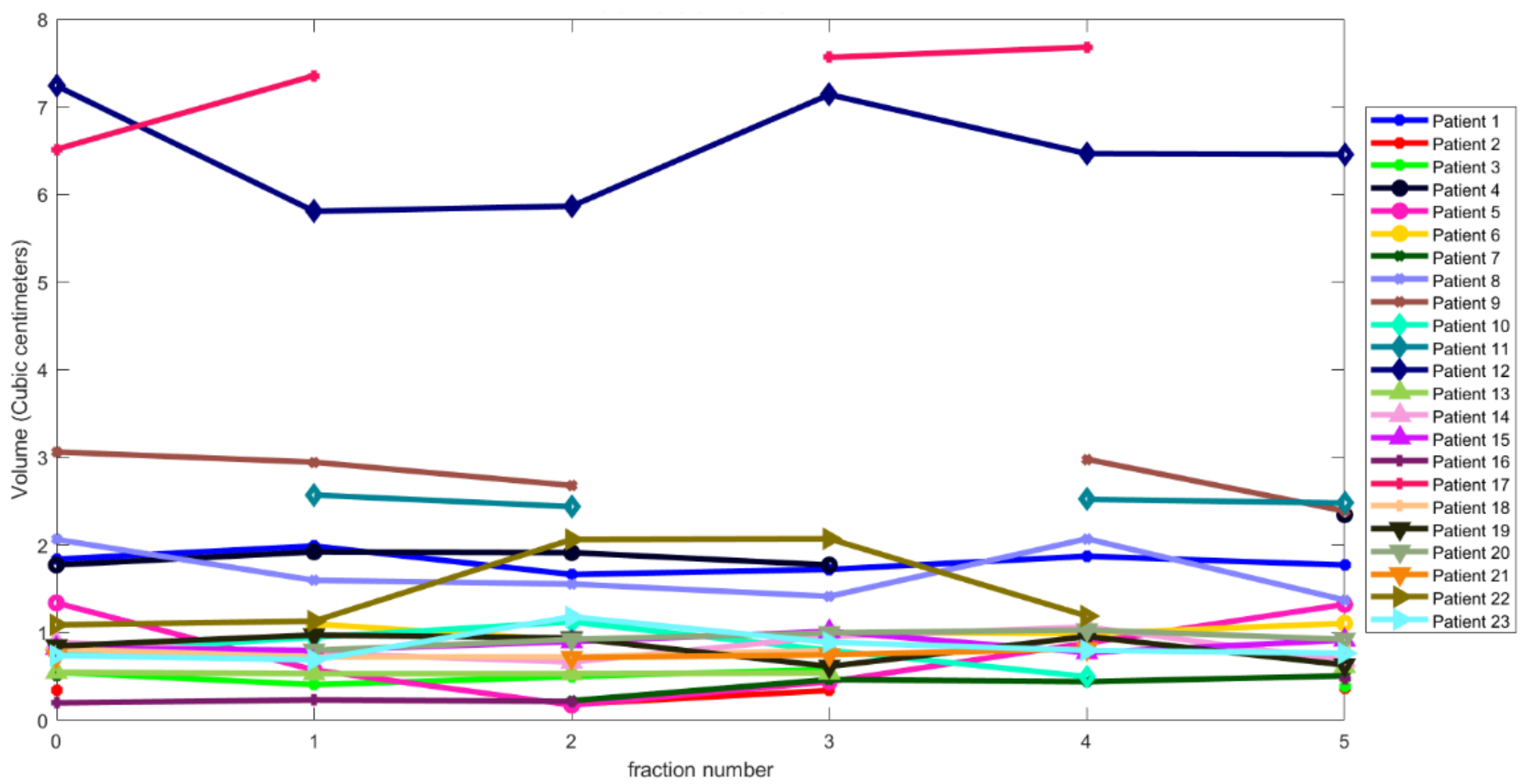

Supplement: Supplementary file 1 [file mmc1.pdf]
